# Heterogeneous repolarization creates ventricular tachycardia circuits in healed myocardial infarction scar

**DOI:** 10.1038/s41467-022-28418-1

**Published:** 2022-02-11

**Authors:** Kamilla Kelemen, Ian D. Greener, Xiaoping Wan, Shankar Parajuli, J. Kevin Donahue

**Affiliations:** 1 Heart and Vascular Research Center, MetroHealth Medical Center, Case Western Reserve University, Cleveland, OH USA; 2 Division of Cardiology, University of Massachusetts Medical School, Worcester, MA USA

**Keywords:** Ventricular tachycardia, Cardiomyopathies, Mechanisms of disease

## Abstract

Arrhythmias originating in scarred ventricular myocardium are a major cause of death, but the underlying mechanism allowing these rhythms to exist remains unknown. This gap in knowledge critically limits identification of at-risk patients and treatment once arrhythmias become manifest. Here we show that potassium voltage-gated channel subfamily E regulatory subunits 3 and 4 (KCNE3, KCNE4) are uniquely upregulated at arrhythmia sites within scarred myocardium. Ventricular arrhythmias occur in areas with a distinctive cardiomyocyte repolarization pattern, where myocyte tracts with short repolarization times connect to myocytes tracts with long repolarization times. We found this unique pattern of repolarization heterogeneity only in ventricular arrhythmia circuits. In contrast, conduction abnormalities were ubiquitous within scar. These repolarization heterogeneities are consistent with known functional effects of KCNE3 and KCNE4 on the slow delayed-rectifier potassium current. We observed repolarization heterogeneity using conventional cardiac electrophysiologic techniques that could potentially translate to identification of at-risk patients. The neutralization of the repolarization heterogeneities could represent a potential strategy for the elimination of ventricular arrhythmia circuits.

## Abstract (web summary)

Ventricular arrhythmias after heart attack are a leading cause of death. Here the authors show, in a porcine model, that KCNE3 and KCNE4 upregulation and a unique pattern of repolarization heterogeneity in the scar facilitate reentrant ventricular tachycardia.

## Introduction

Cardiac arrest is a leading cause of death worldwide^1,2^. Prior investigations have shown evidence of healed myocardial infarction scar in half of cardiac arrest victims^3^. The scar contains a substrate that supports reentrant ventricular arrhythmias, and death is frequently caused by ventricular tachycardia (VT) originating in the infarct scar border^4^. The underlying cellular and tissue electrophysiology that allow reentrant VT to exist in the infarct borderzone is unknown. Histological studies confirm the existence of surviving myocardial tissue strands interspersed with fibrosis in the borderzone^4^, and immunohistochemical studies show decreased and detargeted connexin-43 expression throughout the infarct scar^5,6^, implicating electrical conduction as a component of the arrhythmia mechanism. A problem with ascribing causation of VT entirely to these previously identified factors is that they occur diffusely throughout the borderzone and they are constantly present^5,7^. In contrast, VT occurs relatively rarely, and it exists in discrete circuits confined to specific areas within the scar^4^. If impaired or delayed conduction were sufficient for causation, VT should occur frequently and everywhere in the infarcted myocardium, but it does not. Additional factors must be required for existence of VT at specific times in discrete areas. Identification of the VT mechanism is critical to development of diagnostics and therapeutics for patients at risk for sudden cardiac death.

Here we show that the voltage-gated potassium channel accessory-subunits KCNE3 and KCNE4 are increased uniquely in VT circuits in healed infarct scar. Through interactions with the potassium voltage-gated channel subfamily Q member 1 (KCNQ1), KCNE3 increases and KCNE4 decreases the slow component of the delayed rectifier current, I_Ks_. The functional equivalent of these expression changes is a specific pattern of repolarization heterogeneity in the VT circuit that creates the substrate for reentrant VT.

## Results

### Expression changes in VT circuits within healed infarct scar

**Fig. 1:**
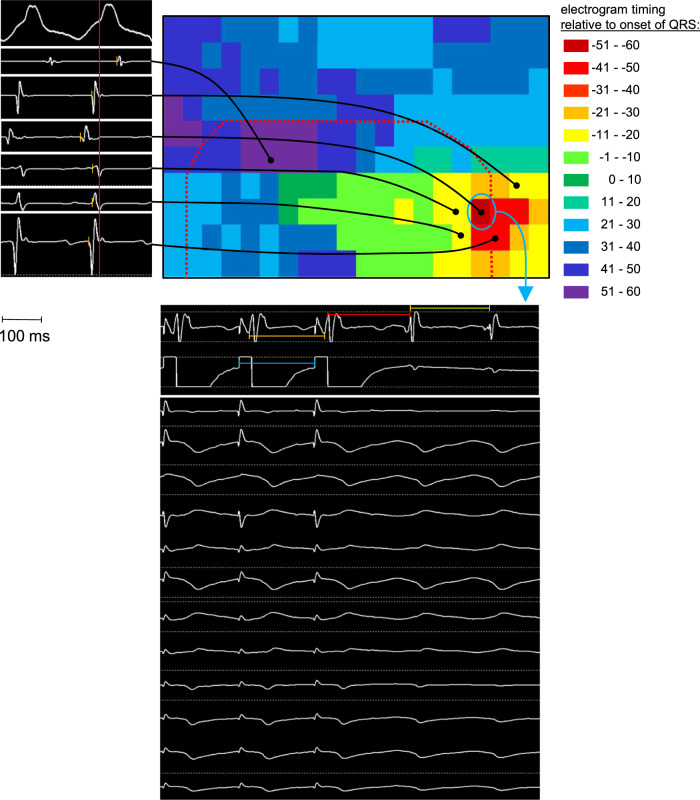
The schematic depicts the VT mapping process, including example electrograms (top left), a graphical display of the activation time during VT (top right with markers showing the location of the example electrograms), and entrainment pacing from the site of earliest activation (local electrograms in the middle panel and 12-lead ECG at bottom). The activation mapping process started by moving the catheter throughout the heart during VT, recording electrogram and marking catheter location at each site, and measuring the timing of each electrogram (top left panel, orange lines) against a fiduciary point in the surface ECG (red line in the top left panel). The activation time and location for each point was displayed graphically (top right). Overlaid on the activation map is the edge of the infarct scar (red dotted line) obtained from a separately performed sinus rhythm voltage map. After identifying earliest activation (blue circle in top right panel), we placed the catheter at that location and paced during VT at an output just above threshold and a cycle length 10 ms faster than the tachycardia cycle length. Local electrograms were recorded from the pacing electrodes and the closest adjacent bipole (middle panel). Local capture was confirmed by comparing the timing of the pacing stimulus (blue bar = 180 ms) to the adjacent electrogram (orange bar = 180 ms). Concealed entrainment criteria were met if the timing of the first return beat after pacing (red bar = 196 ms) was within 20 ms of the tachycardia cycle length (yellow bar = 190 ms), and if the paced QRS morphology exactly matched the VT morphology (bottom panel).

**Fig. 2:**
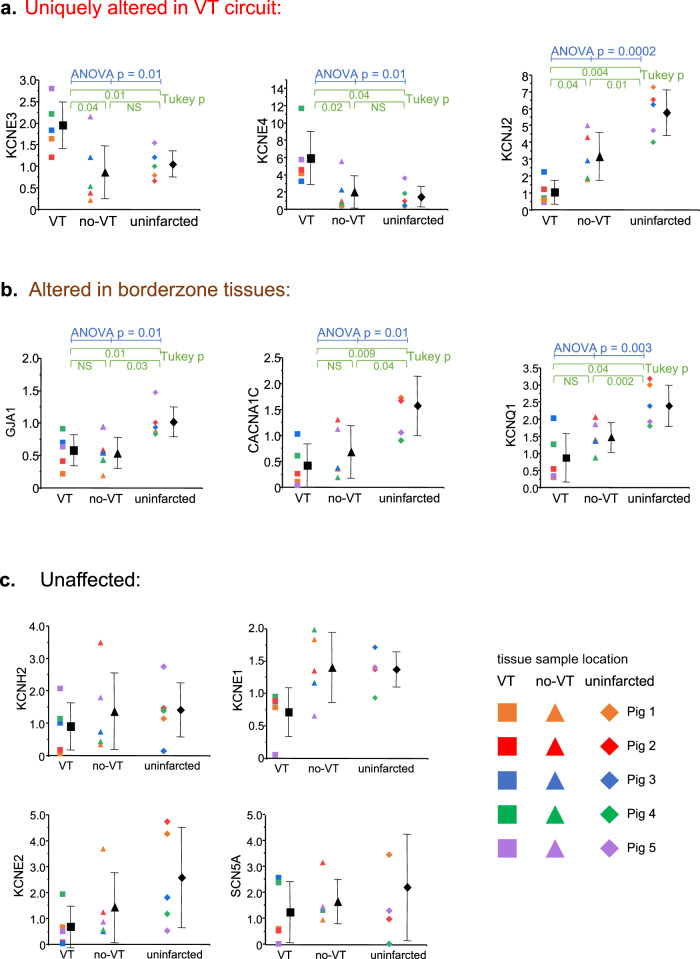
Comparison of mRNA expression of the principle cardiac ion channels and connexin 43 between the mapped VT site (square), a site harvested on the opposite side of the infarct scar from the VT site (triangle) and uninfarcted basal lateral myocardium (diamond). Three patterns of expression emerged in the analysis. **a** Transcripts uniquely altered in the VT circuit. **b** Transcripts decreased generally in borderzone but not uniquely in VT circuits. **c** Transcripts that were not statistically different between the three tested sites. As noted in the text, for these experiments, *n* = 5 biologically independent animals. Mapped VT circuit sites are noted by square symbols, infarct scar tissue not involved in VT by triangle symbols, and uninfarcted myocardial sites by diamond symbols.  Data are reported as mean ± standard deviation. Data analysis included the Shapiro–Wilk test for normality followed by one-way ANOVA with the post-hoc Tukey test to assess differences. Source data are provided as a Source Data file.

To identify elements unique to VT circuits, we first mapped VT (Fig. 1) and then assessed gene expression and electrophysiological function, comparing VT circuits to non-VT scarred myocardium and healthy remote myocardium in a validated, clinically relevant porcine model of post-infarct VT^8,9^. In an initial cohort of five pigs with mature scars after anterior-septal left ventricular myocardial infarctions, we evaluated VT circuit expression for key determinants of cardiac conduction and repolarization. To accomplish this, we identified and harvested VT circuit tissues and compared mRNA expression of the VT circuit to non-VT borderzone harvested from the opposite side of the scar and to uninfarcted myocardium from basal-lateral left ventricle. We identified VT circuit locations using established clinical electrophysiology techniques of activation and entrainment mapping^10,11^. In each animal from this portion of the study, we saw only a single VT morphology per animal, and that morphology was repeatedly and reliably induced with programmed electrical stimulation techniques. After activation mapping to identify a suspected VT circuit, entrainment mapping criteria were tested and fulfilled to identify the protected isthmus where diastolic activation occurred^10,11^. We harvested the VT isthmus in each animal, using a liquid nitrogen-cooled cork borer to insure uniformity in tissue extraction across animals and samples, and we compared mRNA expression between VT site, non-VT borderzone, and uninfarcted myocardium (Fig. 2). We found that potassium channel accessory-subunits KCNE3 and KCNE4 were uniquely upregulated and the potassium inwardly rectifying channel subfamily J member 2 (KCNJ2) was downregulated at VT sites, implicating these ion channel subunits in the VT mechanism. We observed uniform reductions in borderzone expression of gap junction protein alpha 1 (GJA1 encoding connexin 43), calcium voltage-gated channel subunit alpha1 C (CACNA1C), and KCNQ1 at all borderzone sites relative to uninfarcted myocardium, indicating that these were altered diffusely throughout borderzone but not uniquely inside VT circuits. There were no significant differences between subgroups in expression levels of the potassium voltage-gated channel subfamily H member 2 (KCNH2), the potassium voltage-gated channel subfamily E regulatory subunits 1 or 2 (KCNE1, KCNE2) or the sodium voltage-gated channel alpha subunit 5 (SCN5A), suggesting that these ion channels were unaffected by infarction.

### Functional alterations in VT circuits

To identify unique functional elements of VT circuits, we studied a second cohort consisting of ten animals with reproducibly inducible post-infarct VT and five animals with healed infarcts but absolutely no VT on repeated weekly in vivo testing and post-sacrifice ex vivo evaluation. We interrogated functional differences using in vivo bipolar electrogram and monophasic action potential (MAP) recordings, ex vivo optical mapping of perfused cardiac ventricular tissue wedges, and patch clamp analysis of isolated ventricular myocytes from the infarcted pig hearts.

**Fig. 3:**
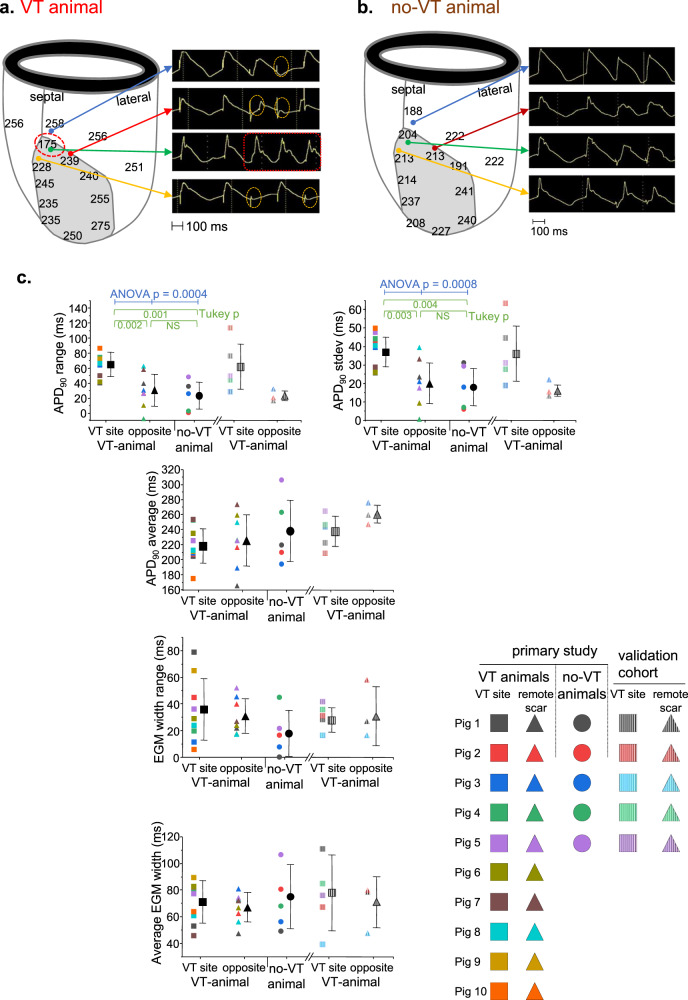
Electrogram analysis from epicardial MAP and bipolar electrogram recordings during in vivo electrophysiology study. **a** APD90 at the indicated sites is shown in the left panel for a single VT animal. The gray region is the infarct scar. The red dashed circle is the location of the mapped VT circuit. The right panel shows MAP recordings illustrating the response to abrupt shortening of the pacing cycle length. Heterogeneity in response to the faster stimulus occurs with sites having conduction delay or block (orange circles) adjacent to a site with continued conduction (red box). **b** A similar map from a no-VT animal shows more homogeneous APDs and uniform conduction with abrupt pacing rate change. **c** Summary data comparing APD and bipolar electrogram width. The analysis from the VT animals included four adjacent electrograms each from the mapped VT site (square) and a site on the opposite side of the infarct scar from the VT site (triangle). In the no-VT animals (circle), we used four adjacent electrograms from a site anatomically matched to the VT site in the VT animals. *n* = 10 biologically independent animals in the VT group, five biologically independent animals in the no-VT group, and five biologically independent animals in the validation cohort. Data are reported as mean ± standard deviation. Data analysis included the Shapiro–Wilk test for normality followed by one-way ANOVA with the post-hoc Tukey test to assess differences. Source data are provided as a Source Data file.

In vivo assessment of action potential duration (APD) on MAP recordings showed a consistent pattern of relatively shorter APDs adjacent to relatively longer APDs at VT circuit locations in the VT animals (Fig. 3a). The no-VT animals had no discernible APD pattern and less overall APD heterogeneity across the infarct scar border (Fig. 3b). The functional consequences of these localized APD heterogeneities became apparent with abrupt change in the pacing rate; the areas with shorter APDs continued to conduct each paced beat after pacing cycle length switched from 400 to 250 ms (red box in Fig. 3a), but the areas with longer APDs had transient conduction delay or block at the faster cycle length (orange circles in Fig. 3a). Transient conduction block in a myocardial tissue tract connecting to another tract with continued conduction is a classical condition for reentry^12,13^. We assessed for conduction heterogeneity in the infarct borderzone by measuring electrogram width and previously described electrogram characteristics of split, fractionated and low amplitude signals and found nothing unique at the VT site compared to non-VT scar in the VT animals or to anatomically paired sites in the no-VT animals. Conduction was impaired throughout the infarct scar border in all animals, suggesting that slow, heterogeneous conduction was a ubiquitous borderzone property. We quantified these observations across all animals (Fig. 3c), and we found no between-group differences in average APD or measures of electrogram width. The APD range and standard deviation were significantly greater at the VT sites, verifying that increased APD heterogeneity occurs specifically at sites where VT was found (Fig. 3c).

After completing the in vivo study in the second cohort of animals, we harvested the hearts and subdivided the infarct borderzone region into myocardial tissue wedges for optical mapping of electrical activity and for cell isolation and patch clamp analysis. In the VT animals, since optical mapping allowed identification and assessment of VT circuits, myocardial tissue wedges used in the optical mapping experiments included both those with and without in vivo mapped VT circuits. Since patch clamp analysis did not afford the possibility of mapping and identifying VT circuits, we only used tissue wedges for cell isolation and patch clamp analysis that came from regions where a VT circuit was mapped in vivo. In the no-VT animals, wedges came from the same anatomical locations as those taken from the VT animals.

**Fig. 4:**
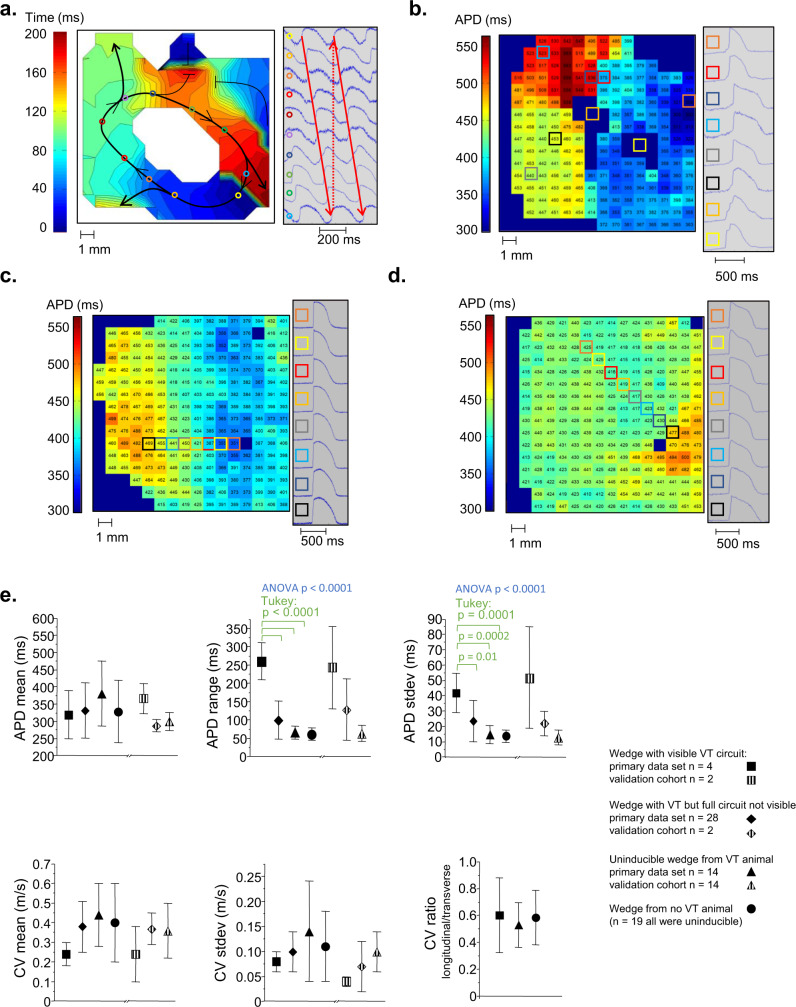
Optical mapping of myocardial borderzone tissues. **a** The entire VT circuit was visible along a single tissue surface in four animals from the primary study and two additional animals in the validation cohort. An isochronal map of a complete VT circuit from one animal is shown. The colored circles indicate locations on the activation map where example pixels at right were located. The white central region had 2:1 activation and did not participate in the VT. **b** An APD map during 1000 ms fixed rate pacing of the full VT circuit tissue from (**a**). The colored boxes show locations on the APD map for the example electrograms at right. All observed VT circuits had tissue with short APDs in contact with long APD tissue. The area marked by the yellow and orange squares had complex, multicomponent activation so APD could not be measured. The APD map from tissue with inducible VT but not the complete circuit (**c**) and the APD map from a no-VT animal (**d**) are shown). **e** Summary data are reported as mean ± standard deviation. Data analysis included the Shapiro–Wilk test for normality followed by one-way ANOVA with the post-hoc Tukey test to assess differences. Source data are provided as a Source Data file.

In the optical mapping experiments, we identified complete VT circuits along single surfaces of tissue wedges in four VT animals (two from the transmural surface of LV free wall wedges, one from the transmural surface of the ventricular septum, and one from the LV free wall endocardium). All of the observed VT circuits had a tract of cells with short APDs (average across all animals with full VT circuits: 228 ± 835 ms) adjacent to a tract with long APDs (496 ± 65 ms) during steady rate pacing that corresponded to the two limbs of the reentrant circuit during VT (Fig. 4a–b). The pattern of distinct short APD (103 ± 25) and long APD (233 ± 54) tracts persisted during VT. In the remaining 42 optical maps obtained from borderzone tissue wedges in VT-group animals, 28 came from wedges that had inducible VT, and 14 came from wedges without inducible VT. In the wedges where VT was induced but the full circuit was not visible (Fig. 4c), 11 maps had focal areas of either shorter or longer APDs but not the connected short-long APD pattern observed in the VT circuits, 1 had progressively increasing APD from the endocardial to the epicardial edges and 16 had no APD gradients. In the 14 maps from uninducible wedges in VT group animals, we saw no APD gradients at all. Similarly, in the 19 optical maps from infarct borderzone tissue wedges for the no-VT group, we saw no APD gradients (Fig. 4d). To quantify the APD heterogeneity in the optical mapping data, we assessed the range and standard deviation of APD measurements across each mapping field. Both measures were significantly longer in the mapping fields with VT circuits (Fig. 4e).

In contrast to the repolarization changes that were unique to VT circuits, conduction velocity was impaired in all infarct borderzone wedges of all animals, and there were no significant differences when comparing overall conduction velocity or measures of conduction heterogeneity or anisotropy between fields with complete VT circuits, incomplete VT circuits or non-inducible wedges in the VT animals or in comparison to the infarct borderzone wedges in the non-VT animals (Fig. 4e).

**Fig. 5:**
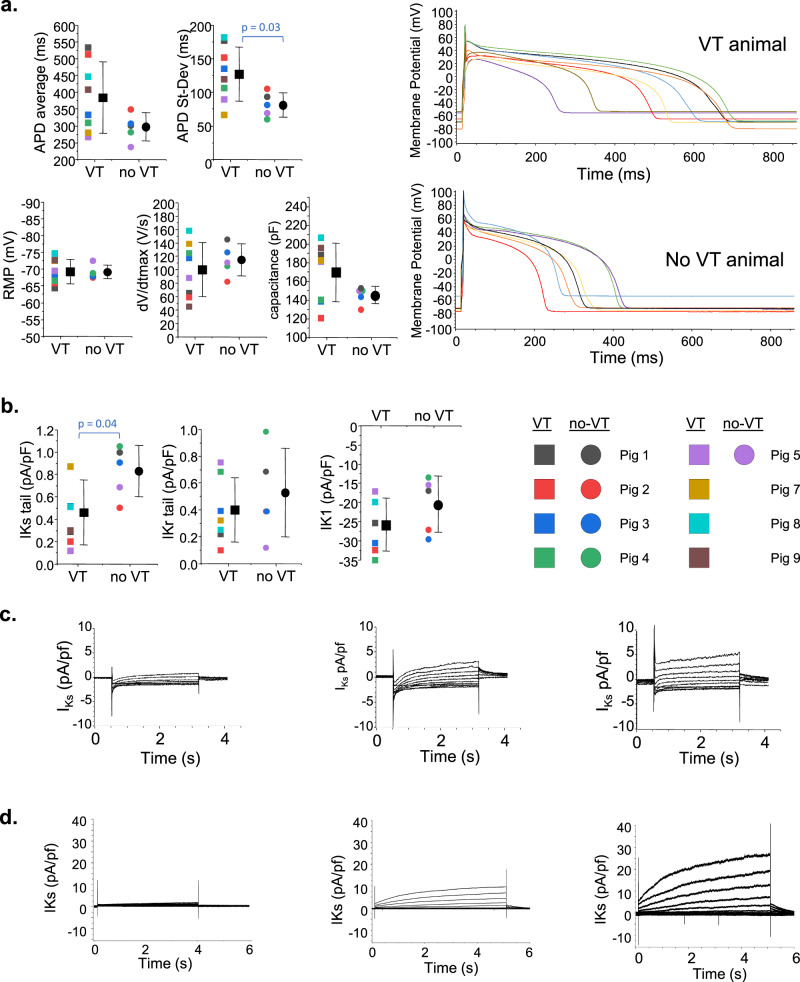
Patch clamp analysis of cardiac myocytes isolated from the VT region in VT animals and from paired anatomic sites in no-VT animals. (**a**) Action potentials show greater heterogeneity in APD for the VT site but no difference in average APD, RMP, or dV/dt_max_. The right panel shows APDs recorded from all cells in a single VT animal compared to a single no-VT animal. **b** Average potassium currents are shown for the three prominent cardiac ventricular repolarizing currents. For the graphs in (**a** and **b**), *n* = 8 biologically independent animals in the VT group and five biologically independent animals in the no-VT group. Data are reported as mean ± standard deviation. Data analysis included the Shapiro–Wilk test for normality followed by Student’s *t* test to assess differences. **c** Variability of I_Ks_ measured from different cells taken within the VT region from a single animal is shown. Some cells had negligible current (left). Other cells had normal current values and morphologies (center), and other cells had increased I_Ks_ with a prominent instantaneous component. This variability was present in 5 of 8 VT animals and 0 of 5 no-VT animals. **d** I_Ks_ tracings from CHO-I_Ks_ cells transfected with KCNE4 (left), control (center), or KCNE3 (right). Source data are provided as a Source Data file.

Unlike the optical mapping findings where we could visualize and measure directly from different parts of the VT circuits, our patch clamp analyses could only come from myocytes that were harvested from the general area where VT circuits were found. We did not have the technology to isolate myocytes only from within the VT circuits. We isolated viable myocytes from myocardial wedges of 8 out of the 10 animals with VT and all 5 animals without VT. In myocytes isolated from the general region of the VT circuits, we found that APDs were more heterogeneous in the VT animals compared to myocytes isolated from anatomically similar areas of the no-VT animals (Fig. 5a). We saw no significant differences between groups in resting membrane potential (RMP), maximum rate of rise of the action potential upstroke (dV/dt_MAX_) or cell capacitance. The repolarizing potassium current *I*_Ks_ was reduced in the VT animals compared to the no-VT animals (Fig. 5b). Other repolarizing potassium currents *I*_*Kr*_ and *I*_K1_ were not significantly different between groups. The lack of between-group differences in RMP or *I*_*K1*_ suggest that the molecular changes observed for KCNJ2 at the VT site may not have functional implications beyond the general downregulation and reduced RMP at all borderzone sites.

In looking at individual current tracings (Fig. 5c), 5 of the 8 VT animals had noticeably heterogeneous *I*_*Ks*_, with some cells having increased *I*_Ks_ with an atypical instantaneous component to the current, some cells having relatively normal *I*_*Ks*_ amplitude and morphology, and other cells from the same myocardial region having decreased or non-existent *I*_Ks_. In the remaining 3 VT animals, we saw only the reduced *I*_Ks_ phenotype. By comparison, in the 5 non-VT animals, 1 animal had cells with only the small *I*_Ks_ phenotype and the other 4 had normal appearing *I*_Ks_ size and morphology.

### Reproduction of ion channel findings in an expression system

To more specifically assess the isolated effect of KCNE3 or KCNE4 overexpression on I_Ks_, we transfected CHO cells that constitutively express KCNQ1 and KCNE1 with either KCNE3 or KCNE4 (Fig. 5d). We found that KCNE3 increased I_Ks_ and KCNE4 essentially eliminated I_Ks_ in the CHO-I_Ks_ cells, verifying the ability of KCNE3 and KCNE4 to affect I_Ks_ in ways similar to what we observed in the VT zone cardiac myocytes.

### Reproduction of study findings in a validation cohort

To assess reproducibility of our findings in an entirely separate cohort of animals, we compared the in vivo electrophysiology and optical mapping results from the VT animals in the current study to stored data from a previously reported cohort of VT animals. In Greener et al., we previously reported VT inducibility and conduction velocity results in animals that had a myocardial infarction procedure, a 4-week post-MI sham gene transfer procedure and then 5-week post-MI sacrifice study^14^. For purposes of the current study, we re-evaluated the five sham gene transfer, control animals that had optical mapping data from that previously published study as a way of independently validating the findings derived from our current study animals. Since this was a retrospective assessment in a small group of animals, we did not statistically analyze the results, but we looked for similar repolarization patterns to those identified in the primary cohort for the current study. We found VT circuit-specific repolarization heterogeneity, both in vivo (Fig. 3c) and in optical mapping experiments (Fig. 4e). In this validation cohort, we found two additional wedges with the full VT circuit visible on a single mapped surface of the wedge, and both had tracts of myocytes with long APDs adjacent to tracts with short APDs like the four complete VT circuits identified in the optical mapping component of the primary study.

## Discussion

The current paradigm for post-infarct VT mechanism in chronic scar (the most common scenario for VT in humans) holds that VT circuits exist where channels of surviving myocardial tissue traverse the scar^11,15,16^. Some have observed normal conduction velocity through the circuitous route of these channels^4,17,18^; while others have suggested that functional conduction delay or block is a component of the VT mechanism^19,20^. Repolarization has not been a consideration in these discussions. A fundamental problem with this understanding of mechanism is that surviving strands of myocardium, connexin lateralization and conduction abnormalities are ubiquitous in chronic myocardial infarct scar tissues^5–7,21^, but VT exists in discrete, ablatable circuits^4,22,23^.

Our data compel a thorough revision of the prevailing paradigm for post-infarct VT mechanism. We show that VT circuits in healed scar reproducibly occur in regions of infarct scar border where a surviving myocardial tissue tract with shorter APDs connects to a myocardial tissue tract with longer APDs. The tract with short APDs adapts to abrupt changes in activation rate, allowing continued conduction at faster rates, and the tract with long APDs has transient conduction block when activation rate changes suddenly. While we did not catch the moment of VT initiation in our data, we did reproducibly find behavior consistent with the proposed mechanism in vivo with transient block in the long APD regions and continued conduction in the short APD regions during abrupt change in pacing cycle length from 400 to 250 ms (Fig. 3a). The general concept for this mechanism of reentry was originally reported almost 100 years ago by Schmitt and Erlanger who altered repolarization by manipulating extracellular potassium concentration in strips of normal turtle ventricular tissues^12^. The concept of repolarization playing an independent and defining role in VT circuits has not previously been considered relevant to the mechanism of post-infarct VT.

**Fig. 6:**
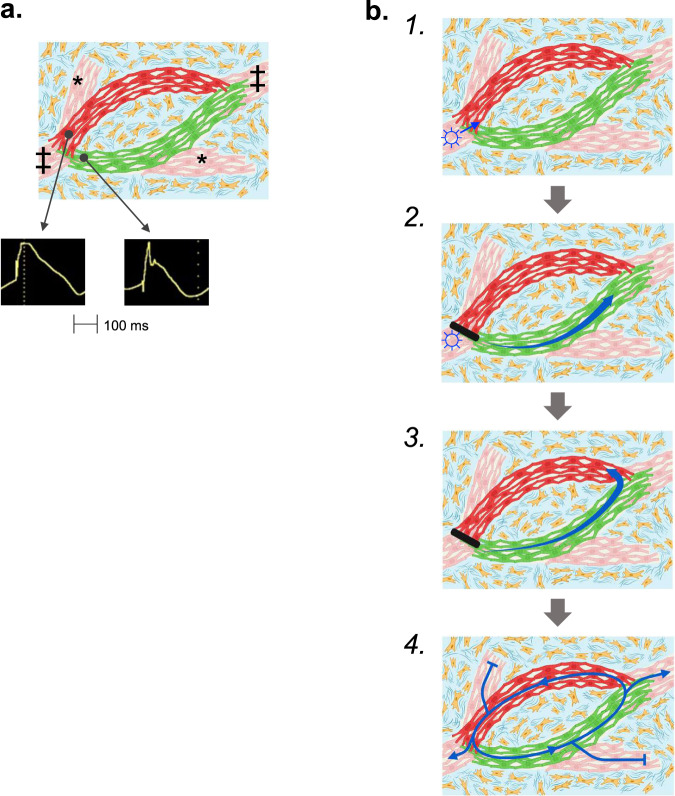
Schematic illustrating the proposed mechanism for VT. (**a**) Basic components of the circuit include surviving strands of myocardium (red, pink and green myocytes) interrupted by areas of fibrosis (blue areas with yellow fibroblasts). A strand of myocytes with shorter APDs (green) is adjacent to a strand of myocytes with longer APDs (red). The circuit connects to the rest of the heart (‡) and may connect to dead-end segments (*). **b** 1. VT starts with a premature beat (☼) that conducts through the surviving myocardial tissue strands until reaching a junction between short and long APD tissues. 2. If appropriately timed, the premature beat continues to conduct down the path with shorter APDs that has recovered excitability (green myocytes) and blocks in the path with longer APDs that is still refractory (red myocytes). 3. When the excitation wavefront reaches the distal connection between the two limbs of the circuit, it continues to conduct back up the long APD limb if that path has recovered excitability. 4. It then continues to conduct in a reentrant manner around the circuit.

Based on these data, we suggest the following paradigm for post-infarct VT mechanism: the basic components of the circuit include a surviving myocardial strand with short APDs from KCNE3 expression linked at proximal and distal points to another surviving myocardial strand with long APDs from KCNE4 expression (Fig. 6a). VT is initiated when a critically timed premature ventricular beat conducts through the many surviving strands of myocardium within the scar to the proximal junction between short and long APD tissues (Fig. 6b1). If appropriately timed, the depolarization wavefront can continue conducting through the myocyte strand with short APDs but it blocks in the still refractory strand with long APDs (Fig. 6b2). If conduction time through the short APD strand is sufficiently long (made more likely by the diffusely impaired conduction throughout myocardial borderzone), the depolarization wavefront would meet excitable tissue at the distal junction between short and long APD tissues, allowing it to conduct back up the long APD strand (Fig. 6b3), and to then continue in a reentrant manner down the short APD strand and back up the long APD strand (Fig. 6b4).

We find that the APD heterogeneity in VT circuits correlates with variability in I_Ks_ current density, caused by upregulation of potassium channel β-subunits KCNE3 and KCNE4. Our observations are consistent with published data on interactions of KCNE3 and KCNE4 with KCNQ1. Ordinarily, KCNQ1 attaches to KCNE1, resulting in the repolarizing potassium current, I_Ks_. Either KCNE3 or KCNE4, when present, can outcompete KCNE1 to bind KCNQ1^24,25^, changing kinetics of the resulting current. When KCNE3 attaches to KCNQ1, it creates a channel with large instantaneous and sustained components relative to what would be expected for normal I_Ks_^24,26,27^. The KCNE4-KCNQ1 interaction abolishes I_Ks_^28,29^. These reported effects of KCNE3 and KCNE4 on KCNQ1 are consistent with the heterogeneous I_Ks_ that we observed in myocytes isolated from the region of VT circuits (Fig. 5c) and the isolated effects of KCNE3 or KCNE4 on CHO-I_Ks_ cells (Fig. 5d). The KCNE3-KCNQ1 currents would create our observed high amplitude I_Ks_ currents and shortened APDs, and KCNE4-KCNQ1 would cause reduction or elimination of I_Ks_ as we observed in other cells from the same area.

Surprisingly, given the importance of the underlying problem, no previously published study of healed MI-VT has mapped the VT and gene expression and function in the VT circuit to non-VT infarct borderzone myocytes to identify unique elements of the VT circuit. The very limited available data generally support the concept of action potential heterogeneity in VT circuits. The only published human data on electrophysiological properties of verified VT circuits comes from de Bakker et al., who identified regions with VT circuits and then explanted the hearts and reported on the action potential characteristics obtained from microelectrode recordings of superfused tissues in the general region where VT was observed^4^. Half of the evaluated myocytes had normal action potentials and the other half had reduced RMPs, slow upstroke velocities, shortened action potential amplitudes, and longer refractory periods. Dangman et al. did not map VT circuits, but they did find prolonged APD and impaired RMP and action potential upstroke in healed MI tissues from hearts extracted at transplant^30^.

No prior animal data have assessed repolarization within VT circuits. Suggestive evidence supporting our observations comes from Dun et al., who reported heterogeneity in I_Ks_ response to adrenergic stimulation in ventricular myocytes harvested 5 days after infarction in dogs^31^. Their data are limited because they did not map VT or attempt to isolate myocytes from the VT circuit, and the infarct scar was in a dynamic state of change during the early stages of healing at the time of their assessment. In a small percentage of cells, Dun found a fast activating, large amplitude current after adrenergic stimulation that was similar in character to our observed baseline I_Ks_ in some of our VT animal myocytes. In Jiang et al. the same group had previously shown a reduction in the KCNE1:KCNQ1 ratio at this post-infarction timepoint^32^, and attributed their observations to KCNQ1-alone channels. They did not assess for KCNE3. Taken together with our data, the findings of Dun and Jiang suggest that the repolarization pattern supporting VT may be established very early after the infarction.

Hegyi et al. investigated cellular electrophysiology in pigs 5 months after small, heterogeneous infarctions caused by microsphere infusion into the first diagonal branch of the left anterior descending (LAD) coronary artery^33^. They did not assess VT inducibility or identify VT circuits. They used a sequential dissection method of patch clamp analysis and found increased late sodium current and leak from the ryanodine receptor in scar borderzone, both of which have been associated with increased triggered arrhythmias. These could potentially explain the ectopic activity that would start a monomorphic VT run. They did not see changes in I_Ks_ in their assessment that did not include interrogation of any VT circuits. Taken together with our data, Hegyi’s data suggest that the premature beat initiating VT could come from anywhere in the scar border, but that the changes in I_Ks_ supporting reentry are likely unique to the VT circuit.

Our data identify a unique and potentially exploitable element of VT circuits in hearts with healed infarct scars. Our proposed mechanism is consistent with the discrete circuit location and relatively rare occurrence found in human post-infarct VT. Translation of these findings could include development of non-invasive screening methods using ECGi (Medtronic, Minneapolis, MN) or similar non-invasive mapping technologies to identify patients with the characteristic repolarization pattern that would suggest risk for post-infarct VT^34^. These repolarization heterogeneities could be targeted to develop more specific and effective therapies, including better antiarrhythmic drug therapy, KCNE3 and KCNE4-directed gene therapies and repolarization mapping for more effective ablation to eradicate VT.

## Methods

### Overall study protocol

The primary study included a total of 20 Yorkshire pigs (starting weight 25–30 kg, indicating an average age of 12 weeks): 5 animals had molecular analysis of harvested VT circuit tissues (3 females and 2 males) and 15 animals had in vivo assessment of electrophysiology followed by ex vivo optical mapping and patch clamp analysis (8 females and 7 males). All animals underwent an initial myocardial infarction/defibrillator implantation procedure. They had non-invasive electrophysiology study performed weekly thereafter until undergoing a sacrifice invasive electrophysiology study and VT mapping procedure 5 weeks after infarction.

In addition to the prospectively collected data from 20 pigs in the primary study, data from a previously reported cohort of 5 pigs was retrospectively analyzed to validate the observations (2 females and 3 males). Animals underwent the same initial myocardial infarction/defibrillator implantation procedure, had weekly electrophysiology study and a 5-week post-MI sacrifice study. The retrospectively analyzed validation cohort also had a sham gene transfer procedure 4 weeks post-MI where they had anesthesia induction, cut-down access to the right carotid artery and jugular vein (the same vessels used in the infarction procedure), and intracoronary infusion of vascular endothelial growth factor, adenosine, and nitroglycerin in saline. Full details of the handling and treatment for those animals can be found in Greener et al.^14^. The animals used in this study were maintained in accordance with the guiding principles of the American Physiological Society, and the experimental protocol was approved by the Institutional Animal Care and Use Committees at Case Western Reserve University and the University of Massachusetts Medical School.

### Myocardial infarction and defibrillator implantation

Full details of the infarction protocol have been previously published^8,9^. After anesthesia induction and sterile preparation, the right external jugular vein and right internal carotid artery were isolated by cut-down. The carotid arterial sheath was connected to a transducer to monitor blood pressure. A 2.7 Fr balloon catheter was inserted through a Judkins JL 3.5 guide catheter into the middle portion of the LAD, immediately distal to the second diagonal branch. Catheter position within the vessel was verified by infusion of iohexol radiographic contrast dye. The balloon was expanded to 4 atm, and the LAD was occluded for 150 min. Prophylactic vasopressors, positive inotropes, and antiarrhythmic drugs were not given. During infarction, blood pressure was treated by dopamine infusion (2–20 mcg/kg/min), phenylephrine bolus (50–200 mcg) or epinephrine bolus (0.1–0.5 mg) and arrhythmias were treated with amiodarone bolus (150 mg over 10 min), as needed.

An implantable cardioverter-defibrillator (ICD, Boston Scientific, Natick, MA) was inserted into a subcutaneous pocket in the left neck and connected to a high voltage active-fixation lead placed through the external jugular vein into the right ventricular apex under fluoroscopic guidance. The ICD was used for programmed stimulation and, if necessary, defibrillation during electrophysiology study. Back-up pacing was activated but not used in any animal.

After completion of the balloon occlusion, catheters and sheaths were removed, and hemostasis was achieved by ligation of the vessel, and the incision was closed. In the post-operative period, the animals were treated with narcotics and non-steroidal anti-inflammatory drugs for pain management. Daily care consisted of observation for overall well-being, pain control, and gross assessment of input, output, and respiration.

### Electrophysiology study (EPS)

For all studies, ECG and intracardiac electrograms were recorded using the EP-Workmate system (EP MedSystems, St. Jude Medical, West Berlin, NJ).

On a weekly basis, animals underwent programmed stimulation of the ventricles to assess VT inducibility. Non-invasive EPS was performed using the ICD. Prior to non-invasive EPS, animals were sedated, and pacing parameters were measured through the defibrillator. Arrhythmia induction was performed using the programmed stimulation protocol described below. If VT was induced, 10 s of 12-lead ECG recording were obtained with the EP system and then the animal was paced out of the rhythm using the ICD antitachycardia pacing algorithms. If burst pacing failed, the animal was shocked internally from the ICD. If VF was induced, the animal was immediately shocked through the ICD, and if needed from an external defibrillator. Since the ICD lead was fixed in the RV apex, only single site programmed stimulation was performed during the non-invasive EPS.

Invasive EPS was performed immediately prior to sacrifice, 5 weeks after infarction. For all EPS measurements, isoflurane was used at a fixed dose of 1.75%.

In the initial 5 VT mapping/molecular analysis animals, the left carotid artery and external jugular vein were isolated by cut-down and a sheath was placed in each vessel. The chest was opened by median sternotomy. Sinus rhythm voltage mapping, VT induction and VT 3-dimensional mapping were performed using protocols described below. At the completion of mapping, all data were reviewed to verify that activation and entrainment criteria had been met and then the marked VT site was snap frozen with a liquid nitrogen-cooled 0.25” cork borer, freed of the remaining heart tissue, freed from the cork borer, immersed in RNAlater (Qiagen, Waltham MA), and stored initially in liquid nitrogen and later in a −80 °C freezer until used for mRNA measurements.

In the subsequent ten functional assessment animals, the chest was opened by median sternotomy. The pericardial sac was incised to expose the heart. The left carotid artery and external jugular vein were isolated by cut-down and a sheath was placed in each vessel. Monophasic action potential (MAP) and bipolar electrogram recordings were obtained at 1 cm intervals around and through the MI scar border. Borderzone location was established by direct visualization and confirmed by observation of decreased electrogram voltage. MAP recordings were obtained by placing a multipolar electrophysiology catheter perpendicular to the tissue and applying pressure. Monophasic electrograms are recorded with the electrode 3(−) to electrode 1(+) configuration that we and others have shown to reproduce the MAP^14,35,36^. MAP recordings were obtained during fixed rate pacing from catheter poles 2 and 4 for 8 beats at 400 with abrupt switch to 250 ms for an additional 8 beats. At each MAP site, in addition to the MAP signal, bipolar electrograms were recorded during sinus rhythm. Both MAP and bipolar electrogram recordings were low pass filtered at 500 Hz, and bipolar recordings were additionally high pass filtered at 30 Hz, as per convention. MAP recordings had the high pass filter set to DC. The MAP recording was used to measure APD_90_ which was taken by measuring the time from AP upstroke to 90% repolarization. The bipolar electrograms were used to measure electrogram width and to record electrogram characteristics of split, fractionated and low amplitude (<1.5 mV) signals. Bipolar electrogram duration was measured using the paced fragmented electrogram duration technique by measuring the time from initial departure to final return to baseline after high- and low-pass filtering at 100 and 500 Hz, as previously reported^14,37,38^.

After completion of electrogram recordings, programmed stimulation was performed using the protocol described below. If VT was induced, mapping was performed as described below. After completion of the mapping procedure, 10,000 units of heparin was administered intravenously and then the heart was harvested. The coronary arteries were flushed with cardioplegic solution and the heart was stored in ice-cold cardioplegic solution for the 3–5 min required for transport to the optical mapping room.

For arrhythmia induction, the following programmed stimulation protocol was performed from the ICD lead in non-invasive studies, and initially from a quadrupolar catheter placed in either the right (molecular analysis animals) or left (MAP recording animals) ventricular apex for invasive studies. In the invasive studies, after completion of ventricular apical pacing, the catheter was repositioned to the right ventricular outflow track (molecular analysis animals) or left ventricular lateral wall (MAP animals) and the arrhythmia induction protocol was performed at that site. The same stimulation protocol was used in the optical mapping experiments to induce VT in myocardial tissue wedges. All pacing was performed at twice the pacing threshold.

Extra-stimuli were delivered after eight ventricular drive beats (pacing cycle length 250, 300, and 350 ms). The first extra stimulus (S2) was initially set 200–240 ms after the last pacing stimulus of the drive train (S1). S2 was then delivered at progressively shorter coupling intervals, scanning in 10 ms steps until the effective refractory period (ERP) was reached. If no arrhythmias were observed, S2 was reset to a point 30 ms outside the ERP. A second extra stimulus (S3) was then added 170–200 ms after S2 and scanning in 10 ms decrements was repeated until S2 and S3 were both refractory or equal to 140 ms. Again, if no arrhythmias were induced, a third extra stimulus (S4) was similarly introduced and scanning in 10 ms decrements was repeated until S2-4 were refractory or a minimum coupling interval of 140 ms was reached. If VF was induced, the animal was immediately defibrillated from the ICD and from an external defibrillator if ICD shock failed to restore sinus rhythm. If VT was induced, 12-lead ECG recording was obtained and compared to prior VT inductions. The number of different 12-lead ECG morphologies of VT was tracked for each animal. VT mapping was performed as described below.

### VT mapping

3-D electrical activation and voltage mapping was performed in the initial five animals to identify the VT circuit and then harvest that tissue for molecular analysis. The Cardiac Pathways RPM mapping system was used for three-dimensional localization of the VT circuit (Boston Scientific, Natick, MA). Catheters were placed in the right ventricular apex and coronary sinus from sheaths in the jugular venous and in the left ventricle from the carotid sheath. Voltage mapping during sinus rhythm was performed to identify borderzone and dense scar areas by movement of the catheter around the left ventricle and recording the voltage at each position. A minimum of 50 points were collected per animal, with the majority of points focused in the area within and around the infarct scar. Per convention, bipolar voltage < 1.5 mV was considered infarct scar borderzone. After completion of the sinus rhythm map, VT was induced by programmed stimulation. All animals had hemodynamically unstable VT, so mapping consisted of repeated cycles of: VT induction, verification of identical 12-lead morphology for the induced VT, collection of ~10 mapping points over ~30 s, cardioversion and then recovery for 3–5 min. This cycle was repeated until sufficient detail was obtained to visualize VT circuit activation. A minimum of 100 activation points was obtained for each VT circuit map. After completion of the activation map, concealed entrainment criteria were assessed at map locations where mid-diastolic activation was observed. The mapping catheter was placed at these points, pacing threshold was measured, VT was induced, pacing stimuli were delivered at a rate 10–20 ms faster than the VT cycle length and stimulus strength twice threshold. If pacing failed to capture the ventricles, pacing rate and/or output strength were adjusted to achieve capture. After entrainment pacing, the VT was terminated by either burst pacing or shock. The 12-lead ECG morphology during pacing was compared to the VT morphology and the timing of the first post-pacing beat (by convention, called the post-pacing interval—PPI) was compared to the VT cycle length. The VT circuit protected isthmus was considered to be the area that had a PPI within 20 ms of the VT cycle length, an identical 12-lead ECG morphology of entrainment pacing and VT, and mid-diastolic activation. The catheter position at the successful entrainment site was marked with India ink dye injection. At the conclusion of the study, a transmural tissue section at the marked VT location was snap frozen and harvested using a liquid nitrogen-cooled 0.25” cork borer.

For the ten functional assessment animals and the five validation cohort animals, the RPM system was no longer available, so VT site was identified by a combination of endocardial and epicardial activation mapping, performed using an endocardial 64-polar multielectrode basket catheter in the left ventricular endocardium and a decapolar electrophysiology catheter in the left and right ventricular epicardium. Epicardial catheter location was tracked by visualization and endocardial catheter position was determined by comparison to epicardial position marked using a right-angle clamp during fluoroscopy. VT was induced using the programmed stimulation protocol described above. During VT, endocardial electrograms were recorded from the basket catheter and epicardial electrograms were recorded sequentially from 6 to 10 pre-determined epicardial catheter positions equally spaced along the ventricular epicardium. VT was terminated by burst pacing or cardioversion and the site of earliest electrical activation and the progression of activation were noted on the EP recordings. Concealed entrainment was assessed as described above. The schematic in Fig. 1 depicts this manual mapping method.

### Quantitative polymerase chain reaction (qPCR)

Total RNA was extracted by the RNeasy-Mini Kit (Qiagen), and reverse transcription used the Superscript first strand synthesis system (Invitrogen). After reverse transcription, the amount of cDNA was adjusted to 10 ng, and real-time PCR was performed using SYBR green with the Applied Biosystems 7500 Fast Real-Time PCR System (Applied BioSystems) and primers designed using PrimerExpress (Applied BioSystems). Transcripts were normalized to 18S ribosomal RNA. Primers are listed in Table 1.

**Table 1:** qPCR primers.

Gene	Direction	Primer
KCNQ1	Sense	CGTGCGATTCCCCAGAAGAG
	Antisense	AGTCTCCCCTTCCAGGTCCA
KCNH2	Sense	CTGCTGAAGGAGACGGAGGA
	Antisense	TGGCGTTGACGTAGGTGGTG
KCNE1	Sense	ATGGCCCTGTCCAATTCCAC
	Antisense	AGCCTCCAGCTTGCTGTCAT
KCNE2	Sense	GATGCGGAGAACTTCTACTACGTCATC
	Antisense	TCCTCCACGATGTACTGGTGG
KCNE3	Sense	TGCTATGGAGACTACCAATGGGACCGAG
	Antisense	CCGCCGCTCCTCAGTCAGGTG
KCNE4	Sense	TCCTTCTACGGCATTTTCTTGA
	Antisense	CATGGGCAGCGGCTTCATAG
KCNJ2	Sense	GGACCTTACTCTTCCCGTTC
	Antisense	GTGTGAGAACCAACCGCTAC
CACNA1C	Sense	GTGTTCCAGTGCATCACCATGG
	Antisense	GTTGACAGATTCGGTCTCACTTG
SCN5A	Sense	GTCTTCTGCCTCAGCGTCTT
	Antisense	TACGATTGAGCACCGTCAAG
GJA1	Sense	AGGTGGACTGTTTCCTCTCTCG
	Antisense	CGATCCTTAACACCCTTGAAGAAGAC
18S ribosomal RNA loading control:	Sense	GTTGTTGCCATGGTAATCCTGCTCAGTACG
	Antisense	TCTGACTTAGAGGCGTTCAGTCATAATCCC

### Optical mapping of perfused ventricular tissue wedges

The cardiac ventricles were removed from the remaining heart and dissected into LV free wall, septum, and RV free wall sections by cutting along the right and left septal borders. In the LV free wall section, the branches of the LAD and circumflex coronary arteries that were perfusing infarct scar or borderzone territory were identified. Starting with the branch perfusing the basal anterior-septal region (generally the second diagonal branch of the LAD), each branch noted to perfuse a section of infarct scar or borderzone was cannulated and the perfusion territory was dissected free of the remaining LV free wall into an individual myocardial tissue wedge. The LAD was opened and branches perfusing the septal borderzone were identified, cannulated, and dissected into myocardial wedges similar to the LV free wall. The RV was not used. An LV free wall wedge from a region identified in VT mapping to be a VT circuit was used for cell isolation and patch clamp as described below. The remaining tissue wedges were used for optical mapping experiments. Optical mapping tissue wedges were perfused with cardioplegia and stored in ice-cold cardioplegia solution until use.

To perform optical mapping of electrical activity, an individual myocardial tissue wedge was perfused with oxygenated Tyrode’s solution at a perfusion pressure of 50–60 mmHg. Poorly perfused edges of the tissue were trimmed, and open vessel ends were ligated prior to use. The tissue was immersed in 37 °C perfusate and stabilized against a flat imaging window within a plexiglass chamber. The preparation was perfused with the voltage-sensitive dye di-4-ANEPPS (15 µM) for 10 min and then the excitation-contraction uncoupler, blebbistatin (10 μM) for 10–20 min (until contraction ceased). For recordings, the dye was excited with a tungsten-halogen light source (Oriel Instruments, Stratford, CT) through a 514 ± 5 nm bandpass filter. The fluoresced light was high-pass filtered at 610 nm and recorded onto a 16 × 16-element photodiode array (model C4675; Hamamatsu) through high numerical aperture photographic lenses using a tandem-lens configuration (Nikon; 85 mm, F/1.4 and 105 mm, F/2.0). The optical signals were amplified with a variable gain (1×, 50×, 200×, 1000×), filtered with a variable cutoff low-pass filter, AC coupled with a variable time constant (1.8 s, 2.2 s, 10 s, DC). Membrane potentials were recorded with sufficient voltage (1 mV), temporal (0.3 ms) and spatial (0.9 mm) resolution to monitor the time course of the AP simultaneously from 256 sites in a 4.5 × 4.5 mm area (giving 350 μm resolution between recording sites). Recordings of the myocardial wedge surfaces were made during constant rate pacing at 1000 ms cycle length. After constant rate pacing, programmed stimulation was performed using the protocol described above to induce VT. If VT was induced, a recording was made of each wedge surface during VT.

Recorded signals were processed using a previously published and validated algorithm that marked activation (50% of peak) and repolarization (90% from peak back to baseline) time points for each pixel^14,35,39,40^. Conduction velocities were calculated from the tissue activation data. APD90 was calculated from activation and repolarization data. VT circuits (if visible) were identified from analysis of the activation pattern during VT.

### Cardiac myocyte isolation and patch clamp analysis

Ventricular myocytes were isolated from sections of myocardium using the following protocol: A wedge of border zone tissue was perfused with Krebs’ buffer for 5 min at 30–40 ml/min, at which time any unperfused segments are removed and open vessels were ligated. The wedge was next perfused with nominally calcium-free solution of the following composition (in mM): 130, NaCl; 5.4, KCl; 2 MgSO_4_; 0.5 NaH_2_PO_4_; 10, glucose; 5, HEPES; 20, Taurine; pH 7.2–7.4 with 100% O_2_; 37 °C for 2.5 min. Perfusion was then switched to digesting solution into which 0.05 mM Ca^2+^, 1.5 mg/ml collagenase (Type II, Worthington), 0.1 mg/ml protease (fraction XIV, Sigma) were added. This solution was then recirculated for ~55 min at 10 ml/min. After digestion, the tissue was minced with HK Solution of the following composition (in mmol/L): l-glutamic acid monopotassium salt 110; KCl 25; MgCl_2_ 3.5; KH_2_PO_4_ 10; glucose 20; HEPES 5; taurine 20; EGTA 0.5; creatine 5; pH 7.2 and filtered into culture tube through a 200 μm nylon mesh filter. Two hours later myocytes were centrifuged and stored with Medium 199 and kept at room temperature until use.

For analysis, only rod-shaped cells with clear striations and no visible blebs were used. Cell viability was monitored throughout the patch protocol and only cells with stable appearance and membrane potentials were used. Isolated myocytes were placed in the Tyrode’s solution composed of (mmol/L) NaCl 137, KCl 5.4, CaCl_2_ 2.0, MgSO_4_ 1.0, Glucose 10, HEPES 10, pH to 7.35 with NaOH. Microelectrodes were fabricated from TW150F borosilicate glass capillaries and filled with a solution of (mmol/L) aspartic acid 120, KCl 20, MgCl_2_ 2, and HEPES 5, NaCl 10, EGTA 5, Na-GTP 0.3, Phosphocreatine 14, K-ATP 4, Creatine phosphokinase 2 and brought to a pH of 7.3. Action potentials and currents were recorded by ruptured-patch technique at 35 °C. Cell capacitance and series resistance were compensated electronically at ~80%. Command and data acquisition were operated with an Axopatch 200B patch clamp amplifier controlled by a personal computer using a Digidata 1200 acquisition board driven by pCLAMP software version 9, and data were interpreted using Clampfit version 9 (Axon Instruments, Foster City, CA).

Action potentials were recorded under current clamp conditions. Myocytes were paced in the current clamp mode using a 1.5 – 2x diastolic threshold, 5 ms current pulse at 1000 ms fixed rate pacing.

I_Ks_ currents were elicited under voltage-clamp conditions from a holding potential of −40 mV with depolarizing voltage pulses from −30 to 60 mV for 2.5 s and then return to −40 mV to generate outward tail currents in the presence of 5 μM E4031 to block I_Kr_. The amplitude of tail currents was normalized to cell capacitance to obtain I_Ks_ current density. The I_Ks_ instantaneous component was calculated as the ratio of initial to peak current.

Measurements of I_Kr_ currents were performed in the presence of 1 μM nisoldipine to block L-type Ca currents. I_Kr_ currents were elicited from a holding potential of −40 mV with depolarizing voltage pulses from −30 to 60 mV for 750 ms and were isolated as E4031-sensitive tail current component upon return to −40 mV. The amplitude of E4031-sensitive tail currents was normalized to cell capacitance to obtain I_Kr_ current density.

I_K1_ currents were elicited from a holding potential of −40 mV with depolarizing voltage pulses from −100 to 40 mV for 150 ms, with 1 μM nisoldipine to block L-type Ca^2+^ currents. Peak current amplitude was normalized to cell capacitance to obtain I_K1_ current density.

### KCNE3 or KCNE4 transduction and patch clamp of CHO-I_Ks_ cells

CHO-I_Ks_ cells were given to us by Al George (Northwestern University). These cells constitutively express KCNQ1 and KCNE1. They were grown in Dulbecco’s Modified Eagle’s Medium supplemented with 10% fetal bovine serum and 600 mcg/ml hygromycin to maintain selective pressure for the KCNQ1/KCNE1-expressing cells.

Plasmids containing KCNE3 or KCNE4 were obtained from Origene (Rockville MD). Each gene was cloned into expression plasmids containing the cytomegalovirus immediate/early promoter, and independent ribosomal entry site and reporter gene using conventional methods. Expression plasmids were obtained from AdGene (Watertown, MA). The KCNE3-containing plasmid had the green fluorescent protein reporter, and the KCNE4-containing plasmid had red fluorescent protein. Gene identity was confirmed by sequencing. Expression was confirmed by qPCR prior to use.

Cells were transfected with TransIT-X2 and the indicated plasmid, following manufacturer’s directions (Mirus, Madison, WI). Control cells were exposed to the transfection reagents but no plasmid. Conventional whole-cell voltage-clamp recordings were performed after 48–72 h after transfection. Only green cells for KCNE3 lentivirus infected dish and red cells for KCNE4 lentivirus infected dish (when viewed under fluorescence microscope) were used for patch-clamp recordings. I_Ks_ currents were elicited by holding cells at −40 mV and then depolarized from −40 to +50 mV in the interval of 10 mV for 5000 ms and then repolarized back to −30 mV to get tail currents.

### Statistical analysis

All experiments were prospectively designed, except for the validation set where the prospectively designed analyses from the initial cohort were applied retrospectively to stored data from the control (infarcted but no gene transfer) animals that we previously reported^14^. Data were analyzed for normal distribution using the Shapiro–Wilk test and found to be normally distributed. Data are reported as mean and standard deviation, and significance was determined at the 0.05 level after correction for multiple comparisons using the method of Bonferroni. Statistical testing of continuous data between two groups was performed using the two-sided students *t* test, and comparisons involving multiple groups were performed using one-way ANOVA with Tukey’s post-hoc test for between-group assessment.

### Reporting summary

Further information on research design is available in the Nature Research Reporting Summary linked to this article.

## Supplementary information

Reporting Summary

## Data Availability

The data supporting the findings from this study are available within the paper and its supplementary information. Source data are provided with this paper. Any remaining raw data will be available from the corresponding author upon reasonable request. Source data are provided with this paper.

## Source data

Source Data

## Code availability

No proprietary software or code was developed for this study. The software used in the study included: 3D VT mapping: Cardiac Pathways Realtime Position Management System model 8100 (Boston Scientific, Marlborough, MA); Electrogram recording and manual mapping: EP WorkMate recording system, initial recordings in software version 1.0.1, later recordings in software version 4.3.2 (EP MedSystems, Abbott. West Berlin NJ), quantitative PCR analysis using the analysis program included with the Applied Biosystems 7500 Real-Time PCR System (SDS version 1.2); Optical Mapping data was analyzed with the algorithm reported by Rosenbaum et al.^39,40^, Patch clamp data was recorded using pCLAMP software version 9 and analyzed using Clampfit version 9, The Boston Scientific Latitude pacemaker/ICD programmer (model 3120) was used for non-invasive electrophysiology studies, statistical analysis included sample size calculations in the online powerandsamplesize.com program, summary statistics performed in excel 16, assessment of data normality using www.statskingdom.com/shapiro-wilk-test-calculator.html, and ANOVA and post-hoc Tukey calculations using statpages.info/anova1sm.html.
