# Supplementary material for: Heterogeneous repolarization creates ventricular tachycardia circuits in healed myocardial infarction scar
Source: Nat Commun. 2022 Feb 11;13:830. doi: 10.1038/s41467-022-28418-1 (PMC8837660; doi:10.1038/s41467-022-28418-1)
Supplement: Supplementary file 1 — Reporting Summary [file 41467_2022_28418_MOESM1_ESM.pdf]

nature portfolio

Corresponding author(s): Donahue, J. Kevin

Last updated by author(s): Dec 13, 2021

Reporting Summary

Nature Portfolio wishes to improve the reproducibility of the work that we publish. This form provides structure for consistency and transparency in reporting. For further information on Nature Portfolio policies, see our Editorial Policies and the Editorial Policy Checklist.

Statistics

For all statistical analyses, confirm that the following items are present in the figure legend, table legend, main text, or Methods section.

n/a	Confirmed	
☐	☒	The exact sample size (n) for each experimental group/condition, given as a discrete number and unit of measurement
☐	☒	A statement on whether measurements were taken from distinct samples or whether the same sample was measured repeatedly
☐	☒	The statistical test(s) used AND whether they are one- or two-sided<br>*Only common tests should be described solely by name; describe more complex techniques in the Methods section.*
☐	☒	A description of all covariates tested
☐	☒	A description of any assumptions or corrections, such as tests of normality and adjustment for multiple comparisons
☐	☒	A full description of the statistical parameters including central tendency (e.g. means) or other basic estimates (e.g. regression coefficient) AND variation (e.g. standard deviation) or associated estimates of uncertainty (e.g. confidence intervals)
☐	☒	For null hypothesis testing, the test statistic (e.g. F, t, r) with confidence intervals, effect sizes, degrees of freedom and P value noted<br>*Give P values as exact values whenever suitable.*
☒	☐	For Bayesian analysis, information on the choice of priors and Markov chain Monte Carlo settings
☒	☐	For hierarchical and complex designs, identification of the appropriate level for tests and full reporting of outcomes
☒	☐	Estimates of effect sizes (e.g. Cohen's d, Pearson's r), indicating how they were calculated

Our web collection on statistics for biologists contains articles on many of the points above.

Software and code

Policy information about availability of computer code

Data collection	No proprietary software or code was developed for this study. The software used in the study included: 3D VT mapping: Cardiac Pathways Realtime Position Management System model 8100 (Boston Scientific, Marlborough, MA); Electrogram recording and manual mapping: EP WorkMate recording system, initial recordings in software version 1.0.1, later recordings in software version 4.3.2 (EP MedSystems, Abbott. West Berlin NJ), quantitative PCR analysis using the analysis program included with the Applied Biosystems 7500 Real-Time PCR System (SDS version 1.2); Optical Mapping data was analyzed with the algorithm reported by Rosenbaum et al.,(manuscript references 39,40), Patch clamp data was recorded using pCLAMP software version 9 and analyzed using Clampfit version 9, The Boston Scientific Latitude pacemaker/ICD programmer (model 3120) was used for non-invasive electrophysiology studies, stastical analysis included sample size calculations in the online powerandsamplesize.com program, summary statistics performed in excel 16, assessment of data normality using www.statskingdom.com/shapiro-wilk-test-calculator.html, and ANOVA and post-hoc Tukey calculations using statpages.info/anova1sm.html.
Data analysis	No proprietary software or code was developed for this study. The software used in the study included: 3D VT mapping: Cardiac Pathways Realtime Position Management System model 8100 (Boston Scientific, Marlborough, MA); Electrogram recording and manual mapping: EP WorkMate recording system, initial recordings in software version 1.0.1, later recordings in software version 4.3.2 (EP MedSystems, Abbott. West Berlin NJ), quantitative PCR analysis using the analysis program included with the Applied Biosystems 7500 Real-Time PCR System (SDS version 1.2); Optical Mapping data was analyzed with the algorithm reported by Rosenbaum et al.,(manuscript references 39,40), Patch clamp data was recorded using pCLAMP software version 9 and analyzed using Clampfit version 9, The Boston Scientific Latitude pacemaker/ICD programmer (model 3120) was used for non-invasive electrophysiology studies, stastical analysis included sample size calculations in the online powerandsamplesize.com program, summary statistics performed in excel 16, assessment of data normality using www.statskingdom.com/shapiro-wilk-test-calculator.html, and ANOVA and post-hoc Tukey calculations using statpages.info/anova1sm.html.

For manuscripts utilizing custom algorithms or software that are central to the research but not yet described in published literature, software must be made available to editors and reviewers. We strongly encourage code deposition in a community repository (e.g. GitHub). See the Nature Portfolio guidelines for submitting code & software for further information.

Data

Policy information about availability of data

All manuscripts must include a data availability statement. This statement should provide the following information, where applicable:

- Accession codes, unique identifiers, or web links for publicly available datasets
- A description of any restrictions on data availability
- For clinical datasets or third party data, please ensure that the statement adheres to our policy

The data supporting the findings from this study are available within the manuscript or the Source Data File. Source data are provided with this paper.

Field-specific reporting

Please select the one below that is the best fit for your research. If you are not sure, read the appropriate sections before making your selection.

- [x] Life sciences
- [ ] Behavioural & social sciences
- [ ] Ecological, evolutionary & environmental sciences

For a reference copy of the document with all sections, see nature.com/documents/nr-reporting-summary-flat.pdf

Life sciences study design

All studies must disclose on these points even when the disclosure is negative.

Sample size

For the initial study comparing VT site to no-VT borderzone and uninfarcted myocardium, no published data were available to determine effect size. As a reasonable estimate, we used data from Peters et al. Circulation 1993;88:864-75 since it compared infarcted vs. normal myocardium for a parameter relevant to our investgation. They found that gap junction surface area in uninfarcted myocardium was double that measured in infarcted myocardium, suggesting an expectation of a large effect size would be reasonable. Within-group variance was relatively tight at one-third of the group mean, and consistent between groups. Based on these estimates, sample size calculations using the online powerandsamplesize.com program determined that 5 animals would give a power of 0.9 to detect differences with an effect size of 0.8 at a type I error rate of 0.05

For the subsequent study of 15 animals comparing those with and without VT, we focused on differences in APD between groups as the parameter most relevant to the observation of KCNE3 and KCNE4 expression found in the initial study. We also took into consideration our published experience that animals in the porcine infarct-VT model who never had VT would be less common than animals with VT. Using APD data from prior animals (APD mean: 450 ms ± 50 ms), we found that 10 animals in the VT group and 5 animals in the no-VT group would give a power of 0.8 to detect differences with an effect size of 0.8 at a type I error rate of 0.05.

Data exclusions

No animals were excluded. No observations within animals were excluded.

Replication

Experiments were not "replicated." A validation cohort was used to verify the main findings of the experimental group, as described in the manuscript. This was an independent group of animals.

Randomization

Animals could not be randomized because the fundamental property under study (presence or absence of ventricular tachycardia circuits) was naturally occurring and thus not controllable. All animals were treated the same using standard protocols, so animal source, infarct size, pre- and post-infarct treatments were all the same to avoid introducing confounding variables. The CHO-IKs cell transfections were not randomized or statistically analyzed.

Blinding

The endpoint of VT presence or VT circuit presence was not known at the time of data collection so all collection was the same regardless of group. Data analysis was performed by investigators who were blinded to study group identity of the animals at the time of analysis.

For the CHO-IKs cell transfections, blinding was not possible because the same investigator was responsible for transfection and patch clamp (S.P.). To minimize bias, that investigator was kept unaware of the findings of the main study.

Reporting for specific materials, systems and methods

We require information from authors about some types of materials, experimental systems and methods used in many studies. Here, indicate whether each material, system or method listed is relevant to your study. If you are not sure if a list item applies to your research, read the appropriate section before selecting a response.

Materials & experimental systems

n/a	Involved in the study	
☒	☐	Antibodies
☐	☒	Eukaryotic cell lines
☒	☐	Palaeontology and archaeology
☐	☒	Animals and other organisms
☒	☐	Human research participants
☒	☐	Clinical data
☒	☐	Dual use research of concern

Methods

n/a	Involved in the study	
☒	☐	ChIP-seq
☒	☐	Flow cytometry
☒	☐	MRI-based neuroimaging

Eukaryotic cell lines

Policy information about cell lines

Cell line source(s)	The CHO-IKs cell line was provided by Alfred L. George, Jr., MD. Chair, Department of Pharmacology, Northwestern University
Authentication	Continued expression of the IKs current was verified by patch clamp analysis. Other properties of this CHO cell were not authenticated.
Mycoplasma contamination	Mycoplasma contamination was not tested.
Commonly misidentified lines (See ICLAC register)	This cell line is not listed in the register of commonly misidentifed cell lines.

Animals and other organisms

Policy information about studies involving animals; ARRIVE guidelines recommended for reporting animal research

Laboratory animals	The study used Yorkshire pigs (starting weight 25-30 kg). Pig weight indicated an average age of approximately 12 weeks. Exact pig age was not provided by the vendor. Of the 25 total animals in the study, 13 were female and 12 were male.
Wild animals	The study did not involve wild animals
Field-collected samples	No field-collected samples were used.
Ethics oversight	The study was reviewed and approved by the IACUCs at Case Western Reserve University and the University of Massachusetts Medical School

Note that full information on the approval of the study protocol must also be provided in the manuscript.
